# Network Analysis of Gene Transcriptions of *Arabidopsis thaliana* in Spaceflight Microgravity

**DOI:** 10.3390/genes12030337

**Published:** 2021-02-25

**Authors:** Vidya Manian, Jairo Orozco, Harshini Gangapuram, Heeralal Janwa, Carlos Agrinsoni

**Affiliations:** 1 Department of Electrical and Computer Engineering, University of Puerto Rico, Mayaguez, PR 00681-9000, USA; Jairo.orozco@upr.edu; 2 Department of Bioengineering, University of Puerto Rico, Mayaguez, PR 00681-9000, USA; harshini.gangapuram@upr.edu; 3 Department of Mathematics, University of Puerto Rico, Rio Piedras, PR 00925-2537, USA; heeralal.janwa@upr.edu (H.J.); carlos.agrinsoni@upr.edu (C.A.)

**Keywords:** gene expression, graphs, network analysis, root growth, cell wall biosynthesis, spaceflight microgravity, *Arabidopsis thaliana*

## Abstract

The transcriptomic datasets of the plant model organism *Arabidopsis thaliana* grown in the International Space Station provided by GeneLab have been mined to isolate the impact of spaceflight microgravity on gene expressions related to root growth. A set of computational tools is used to identify the hub genes that respond differently in spaceflight with controlled lighting compared to on the ground. These computational tools based on graph-theoretic approaches are used to infer gene regulatory networks from the transcriptomic datasets. The three main algorithms used for network analyses are Least Absolute Shrinkage and Selection Operator (LASSO), Pearson correlation, and the Hyperlink-Induced Topic Search (HITS) algorithm. Graph-based spectral analyses reveal distinct properties of the spaceflight microgravity networks for the Wassilewskija (WS), Columbia (Col)-0, and mutant phytochromeD (*phyD*) ecotypes. The set of hub genes that are significantly altered in spaceflight microgravity are mainly involved in cell wall synthesis, protein transport, response to auxin, stress responses, and catabolic processes. Network analysis highlights five important root growth-regulating hub genes that have the highest outdegree distribution in spaceflight microgravity networks. These concerned genes coding for proteins are identified from the Gene Regulatory Networks (GRNs) corresponding to spaceflight total light environment. Furthermore, network analysis uncovers genes that encode nucleotide-diphospho-sugar interconversion enzymes that have higher transcriptional regulation in spaceflight microgravity and are involved in cell wall biosynthesis.

## 1. Introduction

Gravity plays a key role in plant growth [1]. *Arabidopsis thaliana* (*Arabidopsis*) is a flowering plant that has been chosen by biologists as a model plant organism to study the effects of gravity and other environmental stressors, since it has a short life cycle and because of the existence of a multitude of mutants and transgenic plants. The current visibility of *Arabidopsis* research reflects the growing realization among biologists that this simple angiosperm can serve as a convenient model not only for plant biology but also for addressing fundamental questions of biological structure and function common to all Eukaryotes [2]. To achieve the goal of providing long term life support for deep space missions a fundamental knowledge on plant growth in spaceflight has to be gained. The International Space Station (ISS) is currently equipped with all capabilities to collect data needed to address fundamental questions of plant physiology and development in spaceflight microgravity [3]. 

In this article, we are interested in identifying the genes that show transcriptional abundance under the influence of spaceflight microgravity, and the genes primarily influencing root growth and cell wall biosynthesis in spaceflight. The transcriptomic profile of *Arabidopsis* has been studied in simulated microgravity [4]. The effect of gravity and mechanical stimulation on the growth of the root apex of *Arabidopsis* has resulted in transient changes in gene expressions [5]. The effect of spaceflight microgravity on *Arabidopsis* root growth is presented in [6]. Cell wall modeling in the roots, shoots, and hypocotyls of plants is an important metabolic adaptation brought about by genes and proteins. Metabolic phenotyping of *Arabidopsis* has been carried out on the ground [7]. Metabolic fingerprinting is important for improving the quality of crops grown in spaceflight. Several of the cell wall proteome changes have been corroborated with transcriptomic changes in spaceflight [8]. As plants are important for the bioregenerative life support in spaceflight, the cell wall perturbations may affect the yield of crops grown in the altered spaceflight environment [9]. Of the 33 XTH Arabidopsis root growth genes, XTH17 to XTH20 are the main contributors [10]. Nucleotide sugar interconversions in plants are discussed in [11,12]. From terrestrial studies, nucleotide-diphsopho-(NPD) sugar interconversion enzymes (NSE) of the small dehydrogenase/reductase superfamily are given in [13]. Plant Cell Wall (PCW) genes were identified using gene co-expression analysis in [14], and it was shown how these genes are transcriptionally co-regulated [15]. These sugars require subtle quantitative control during plant root growth and development. Hence, it is important to identify the genes that encode these enzymes involved in regulating growth of adventitious roots, and their waving and skewing in spaceflight microgravity. 

Researchers have used various ecotypes and mutants of *Arabidopsis* to isolate genes with transcriptional variation in spaceflight environment compared to on the ground. Since we are not doing the experiment and performing the data collection, we rely on datasets available from GeneLab for plants grown in spaceflight microgravity. GeneLab provides transcriptomic datasets for various ecotypes of *Arabidopsis* from spaceflight. The *Arabidopsis* ecotypes Col-0, Ws-2, Ler-0, and Cvi-0 showed differential gene expression due to oxidative stress in spaceflight [16]. Differential gene expression analysis of *Arabidopsis* exposed over six days to blue light at different levels of gravity in comparison to unit gravity control has been presented in [17], and it was found that blue-light phototropism may be enough to reduce stress due to lower levels of gravity in spaceflight. It was found that plants in spaceflight microgravity grow slower than plants in unit gravity on the ground. Two ecotypes of *Arabidopsis* (WS and Col-0) were grown in spaceflight under directional light, and it was found that skewing and waving of roots were independent of gravity, the roots were negatively phototropic, and both ecotypes skewed differently in the presence of light, due to a lack of gravity [18]. Major organ-specific and cellular remodeling of *Arabidopsis* has been identified from its growth in ISS and physiological adaptation to spaceflight [3,19]. Previous literature, cited above, characterized unique transcriptions in WS, Col-0, and PhyD mutant of *Arabidopsis* using heat maps and fold change analysis methods. For all the genome analysis done, no phenotypic measurements have been presented or discussed. However, the gradient-light experiments conducted in the ISS resulted in the plant root growth in a skewing pattern. These were dramatic observations of phenotype changes concerning root growth and cell wall response in microgravity. Hence, we have chosen the GLDS-7 [3] and GLDS-120 [20] datasets collected under different lighting settings for applying our network analysis methods. 

The biochemical interactions within a cell of a biological system are formulated as gene regulatory networks for modeling biological processes. GRNs are used to represent regulatory and transcriptional functions that underlie cellular, molecular, and biological phenomena. GRNs have become an indispensable tool that facilitates interpretation of biological processes, molecular functions, protein interactions, genome to phenome analysis, cellular differentiation, identification of genes as biomarkers, and the response of an organism to external stimuli. A Bayesian network generation method for GRN constructed for *Arabidopsis* has been presented [21]. Several machine learning algorithms have been implemented for the generation of these networks [22]. In [23], several statistical and machine learning methods have been reviewed for the prediction of GRNs from transcriptome datasets. In [24], many algorithms for GRN inferencing from single-cell transcriptomic data are benchmarked using simulated data. Several GRNs have been generated to study root development in *Arabidopsis* [25]. Vascular root development has been represented using GRNs in [26]. Furthermore, *Arabidopsis* secondary cell wall synthesis has been studied using a protein-DNA regulatory network and genes involved in secondary cell wall modeling, which form the cell shape and the bulk of the plant biomass [27]. Other GRNs for *Arabidopsis* growth and development are presented in [28]. Gene Ontology is the process of classifying an annotated gene to a particular function. Gene set enrichment analysis is used to determine changes of expression in groups of a priori defined gene sets. Additional web-tools and databases are available for *Arabidopsis* gene expression analysis [29]. These GRN studies do not identify hub genes and the target (authority) genes they regulate in spaceflight microgravity compared to on the ground for important biological processes that are affected in spaceflight, nor perform rigorous graph theoretic network analysis and derive biological interpretations. 

We have used three basic graph-based network approaches in this paper (1) the LASSO, (2) Pearson correlation, and (3) HITS methods. LASSO and Pearson correlation methods are applied to construct GRNs of transcriptional regulations in spaceflight microgravity as well as ground control. Section 2 presents the materials and methods used for GRN inferencing and analysis. In Section 3, we present the results of the GRN analysis of the significant hub genes (identified from Pearson and HITS method) with the highest transcriptional abundance in spaceflight microgravity. Results from network analysis of the *Arabidopsis* ecotypes and genotype are discussed in this section. The root growth and cell wall genes affected in spaceflight microgravity are isolated and their molecular functions are discussed. Section 4 presents the conclusions.

## 2. Materials and Methods

### 2.1. GeneLab Arabidopsis Datasets

We have mined GeneLab GLDS-7 and GLDS -120 datasets for identifying hub genes that are affected by spaceflight. The GLDS-7 dataset contains microarray gene expression data for the *Arabidopsis* WS and Col-0 ecotypes for different tissues (roots, leaves, and hypocotyls) for the plants grown in ground control and spaceflight in white light. GLDS-120 contains the transcriptomics data of the *Arabidopsis* Wassilewskija (WS) and Columbia (Col-0) ecotypes, and the gene *Phytochrome D* (*phyD*) mutant of ecotype Col-0. The different stressors imposed on the plants are 4–6 umoles m-2 s-1 of total light and dark in spaceflight and on the ground. The design of the experiment was to investigate the effect of light on *Arabidopsis* root growth when the gravity stimuli were nullified. The *phyD* mutant was used, since it is a known light-related mutant in Col-0 background. RNAseq is used for transcription profiling. Gene expression data are available for pairs of combinations of genotypes and ecotypes. 

### 2.2. Graph-Based GRN Inferencing

We define a graph-based representation for the gene expressions. Formally, a graph is a pair of sets G = (V, E) where V is the vertices (molecules, genes, proteins, nodes, points) and E is an ordered pair of vertices (u, v) that are called the edges (arcs), respectively [30]. One important advantage of the representation of networks by graphs is that they have an intuitive graphical representation. In a directed graph G = (V, E, o, t), e = (u, v) are the edges, the origin of e is denoted by o(e), and the terminal v is denoted by t(v). A graph is weighted if each edge e is assigned a positive real-valued weight w(e). Principal Component Analysis (PCA) method is applied to de-correlate the gene expression values. PCA is done to reduce the dimensions to three main components corresponding to the largest eigenvalues of the covariance matrix computed from the graph representation of the gene expression data. PCA projects the data on to an orthogonal space, such that the variance of the projected data is maximized. This space is lower in dimension with minimum projection cost. PCA is applied to the gene expression datasets before performing both LASSO regression and Pearson correlation GRN construction.

#### 2.2.1. LASSO Regression

Lasso is a linear regression algorithm that shrinks the data towards a central point. Lasso regression mostly leads to sparse models. Lasso uses the *l_1_* regularization, which means it adds a penalty that is equal to the sum of the absolute value of the coefficients. Lasso regression is computationally feasible and can fit any kind of statistical data [31]. *l_1_* regularization is applied to eliminate the relationship between the genes with the least importance. The Lasso regression is constructed according to the description in [31]. We have used the SILGGM package in R for the Lasso Regression [32]. In this algorithm, every gene present in the data is considered as a transcription factor, as well as a target gene, and the correlation between each gene with the other is calculated using the scaled Lasso algorithm. The results from the regression are used to construct GRNs in Cytoscape for the sub-modules of the genes of *Arabidopsis* spaceflight and ground datasets. The method has been applied to the GeneLab GLDS-120 dataset.

#### 2.2.2. Pearson Correlation 

Pearson’s correlation coefficient between two genes (variables, nodes) is defined as the covariance of the two variables divided by the product of their standard deviations:

(1) $$\rho_{X,Y}=\frac{\mathrm{cov}(X,Y)}{\sigma_{X}\sigma_{Y}}$$

**Figure 1:**
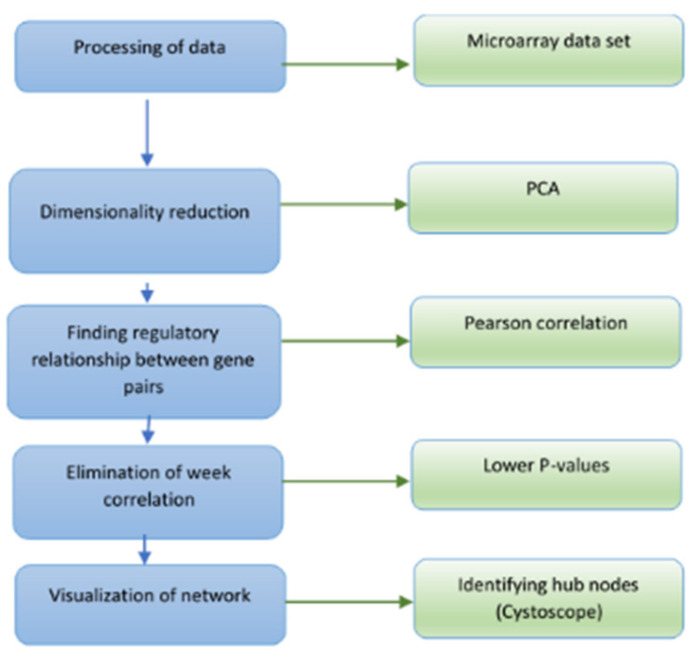
Flow chart of the procedure for gene regulatory network inferencing using the graph-based Pearson correlation method.

Pearson’s correlation coefficient is a test that measures the statistical relationship between two random variables. If the association between the elements is not linear, then the coefficient is not specifically identified. In this study, we use the Pearson correlation to construct the co-expressed gene network to identify genes with correlated expression patterns. This method is based on an adjusted threshold *p*-value to accommodate multiple hypotheses; the correlation between expression patterns is calculated for every pair of genes. The Pearson correlation coefficient is used as the measure of correlation (between −1 to 1) and gene pairs are considered to be co-expressed if their *p*-value is less than or equal to the corrected threshold *p*-value. The *t*-test is used to establish if the correlation coefficient is significantly different from zero, and hence, that there is evidence of an association between the two variables. The genes are treated as nodes and the relation between them is an edge. Therefore, the nodes with the highest degree are related to a higher number of target or authority genes. Those genes that have the highest degree or intra-module (within graph) connectivity are treated as hub genes to construct the gene regulatory networks. Figure 1 shows the steps involved in generating the GRN from the microarray gene expression dataset using this method. 

#### 2.2.3. Sparse Networks and the Weighted HITS Algorithm

We show that the GRN generated for the GeneLab *Arabidopsis* datasets are sparse networks or graphs. We define sparsity precisely so that we utilize quantitatively time and speedups in HITS and other algorithms. Briefly, we recall the following definitions obtained from [30] and [33].

Directed graph: The graph (V, E, o, t) is said to be directed if directed edges are allowed, i.e., not all edges have reverse edges as members of E. In a directed graph, G = (V, E, o, t), the edges e = (u, v), the origin of e is denoted by o, and the terminal v is denoted by t(v).

Bipartite graph: The graph is called *bipartite* if $V=V_{1}⊎\;V_{2}$ such that edges connect node $V_{1}$ to $V_{2}$. The expanding constant or isoperimetric constant (or Cheeger constant) of X is defined as:

(2) $$h\left(X\right)=\;\underset{\emptyset \neq F\subset V}{\min}\frac{\left|\partial F\right|}{\min\left\{\left|F\right|,\;\left|V\backslash F\right|\;\right\}}$$

where F⊂V and the boundary ∂F is the set of edges connecting F to V∖F. The isoperimetric constant is difficult to compute, but we can bound its value using the spectral gap. The Spectral Gap is defined as the difference between the two highest eigenvalues of the adjacency matrix of the graph. 

Sparse graphs: We say that a graph (or the corresponding network) is sparse if $h\left(X\right)$ is large. One of the major results of Cheeger for Riemannian manifolds and its equivalent for graphs is that a large spectral gap implies large $h\left(X\right).$ Computing $h\left(X\right)$ is an NP-Complete problem, while the computing spectral gap is at most cubic and many times linear; we work with the spectral gap here.

Spectral gap: For a graph X, the Laplacian eigenvalues can be ordered as |λ_1_| ≥ |λ_2_| ≥ · · · ≥ |λn| (X may be directed or undirected, weighted or unweighted, simple or not). The spectral gap is defined as: δ_λ_ = |λ_1_| − |λ_2_|. By normalizing the Laplacian matrix of X, the eigenvalues are 1 = λ_1_ ≥ λ_2_ ≥ · · · ≥ λn > 0, and the Lapacian spectral gap will be: δ_λ_ =1 − |λ_2_|. The spectral gap is also known as a random walk, in terms of this concept λ_2_ is the most important eigenvalue. Note that if the spectral gap is 0, λ_2_ = 1 (Γ is not (strongly) connected or Γ is bipartite), and a typical random walk will not converge to a unique distribution or dominant eigenvector. As long as the spectral gap is greater than 0, which means |λ_2_| < 1, then the random walk converges to a unique dominant eigenvector, and the spectral gap measures the rate of convergence; the larger the spectral gap (the smaller |λ_2_|), the better the network flow (large $h\left(X\right)$, diffusion, mixing, random walk, expansion, sparsity, and other highly desirable properties of the network X). 

Girth: The girth of X is the smallest positive integer r such that $Trace\left(A^{r}\right)>0$. Let $d=d\left(X\right)$ be the smallest integer (if it exists), so that for every pair of vertices $\left(u,v\right)$, there is a walk of length at most d from u to v. Then, $d\left(X\right)$ is called the diameter or maximum eccentricity of the graph X.

Clustering coefficient: The clustering coefficient models the degree of clustering of a subset of nodes. A node is selected, and we see how connected the node is with other nodes that are also connected to it. The clustering coefficient is defined by:

(3) $$C_{i}=\frac{L_{i}}{K_{i}\left(K_{i}-1\right)/2}$$

where *L_i_* is the number of connections between $K_{i}$, which are the immediate neighbors of node *i*. The clustering coefficient is used to characterize network modularity, which is a strength of measure of a network division into modules or groups.

Degree distribution: The degree distribution is the number of neighbors connected to a node; in other words, it is the number of edges incident on a node. The degree distribution can give information about the structure of a network. The networks can be directed or undirected. In the undirected case, the degree of a node *i* is the number of connections it has, and it can be represented as an adjacency matrix:

(4) $$K_{i}=\underset{j}{\sum }a_{ij}$$

where the sum is over all the nodes. For directed graphs, there are two types of degree distributions: in-degree, which is the number of connections entering the node, and out-degree, which is the number of outgoing connections. For this case, the sum of the *i*th rows and *i*th columns of the adjacency matrix are:

(5) $$K_{i}^{total}=\underset{j}{\sum }a_{ij}+\;\underset{j}{\sum }a_{ji}\;$$

The degree distribution for a directed graph is the fraction of vertices of degree *k_in_* and *k_out._*

Biomolecular networks such as GRNs follow preferential attachment models, which are scale-free with a degree distribution that follows an exponential law. Unlike the random graph model, these networks have nodes with large degrees, called hubs. Classically, with 0–1 (nonconnection-connection) networks, just the degree distribution is used in the identification of such hubs. A much more sophisticated algorithm is proposed by Kleinberg [34], called the HITS algorithm. Originally, it was meant for networks such as the Internet; however, now, it is used to study a variety of biomolecular networks, such as GRNs and Protein–Protein Interaction (PPI) networks [35,36]. Most of these applications use PageRank to reveal localized information about the graph based on some form of external data. We apply this algorithm in our setting for the weighted and directed networks for the transcription factors-target gene networks and for co-expression networks. 

In the weighted GRN setting, the traditional simplistic method of detecting hub genes would not yield meaningful information. Our approach uses iteratively the weighted HITS algorithm in a novel way as follows. The weighted HITS algorithm computes the hub ${\vec{h}}_{k}$ (resp. authority ${\vec{a}}_{k})\;nodes$ iteratively as: ${\vec{h}}_{k}=\;\psi_{k}\phi_{k-1}AA^{T}{\vec{h}}_{k-2}$ and ${\vec{a}}_{k}=\;\phi_{k}\psi_{k-1}A^{T}A{\vec{a}}_{k-2}$. These iterations converge to the dominant eigenvector of the real symmetric matrices $AA^{T}$ ($A^{T}A$). These give us asymptotically hub and authority weights. A version of this algorithm is implemented and applied to the networks in the software SAGE. Our algorithm gives weighted-hub genes and weighted-authority (target) genes. In complex networks, the HITS algorithm has very high complexity, and cannot be applied successfully. The spectral gap analysis shows that the graphs are sparse but highly connected, helping us carry out HITS with a high degree of iterations. The weighted HITS algorithm has yielded important information about biomolecular networks [37,38,39,40,41,42,43,44,45]. For network topology, we refer to [46], and for the latest on the origin of biomolecular networks, topological, combinatorial, and spectral methods, we refer to [47]. 

## 3. Results and Discussion

**Figure 2:**
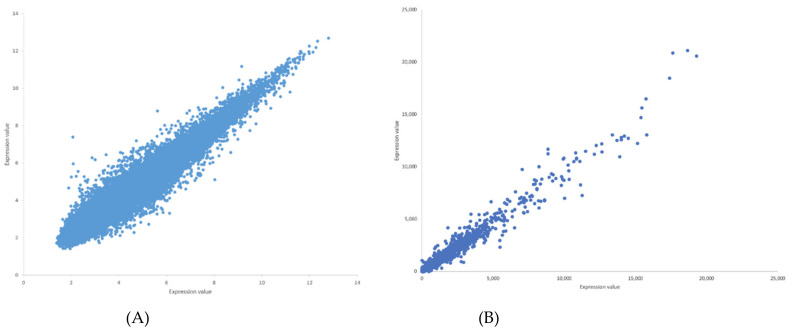
(**A**) Plot of gene expression values for *Arabidopsis thaliana* WS root growth in spaceflight microgravity from GLDS-7 dataset. The plot shows high correlation between the first column (*x*-axis) and second column (*y*-axis) of gene expression values for 20,000 genes. (**B**) Plot of gene expression values for *Arabidopsis thaliana* Col-0 root growth in spaceflight microgravity from GLDS-120 dataset. The plot shows high correlation between the first column (*x*-axis) and second column (*y*-axis) of gene expression values for 20,000 genes.

**Figure 3:**
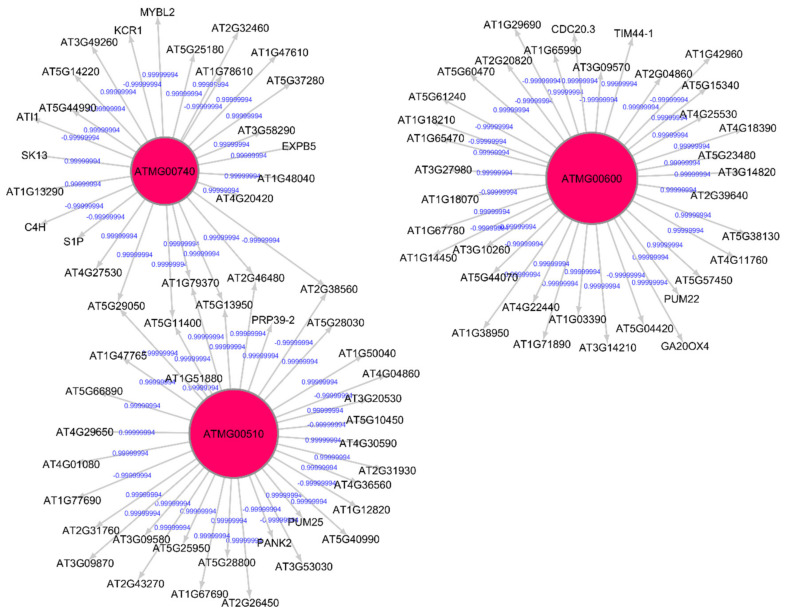
Gene regulatory sub-graph for *Arabidopsis thaliana* root growth in spaceflight microgravity. The hub genes are the red circles representing the strong interactions with differentially expressed genes. The target (authority) genes are shown without circles. The weights on the arrows are the correlation values between the hub gene and the target gene.

**Figure 4:**
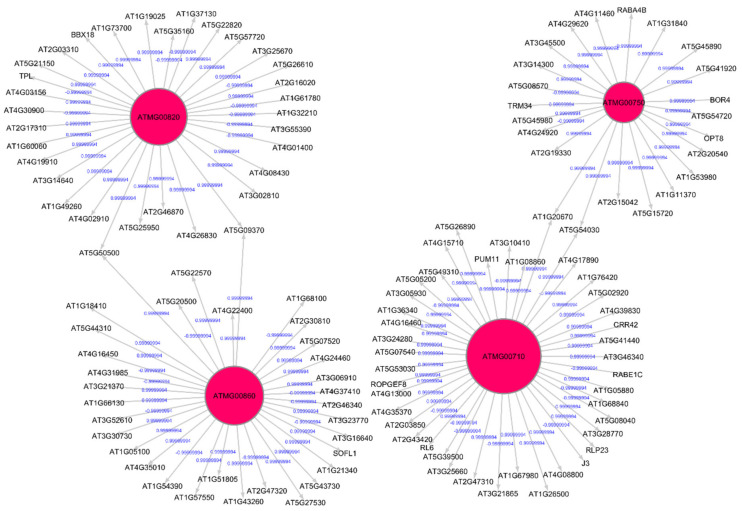
Gene regulatory sub-graph for *Arabidopsis thaliana* root growth in the ground control. The hub genes are the red circles representing the strong interactions with differentially expressed genes. The target (authority) genes are shown without circles. The weights on the arrows are the correlation values between the hub gene and the target gene.

The Pearson correlation method is used to generate weighted directed graphs. Two columns of 20,000 samples of gene expression values of Arabidopsis ecotypes WS and Col-0 root growth in spaceflight from GLDS-7 and GLDS-120 datasets are plotted in Figure 2 (A) and (B), respectively. *X*-axis is the first column of the gene expression values and *y*-axis is the second column of the gene expression values. The plot shows that the values are highly correlated. Hence, PCA is first applied to reduce the dimensionality of the gene expression data to three dimensions. The three PCA components are then used for computing Pearson correlation and generating the network, as explained in Section 2.2.2. All the gene expression values from GLDS-7 and GLDS-120 spaceflight and ground data for Arabidopsis WS ecotype root growth are used for generating the networks. In Table 1, sample correlation values between 13 genes, their t-statistics, and their *p*-values are given. Correlation values range from −1 to 1. *P*-values can also be used to construct GRNs by thresholding them between, for example, 0.01, and 0.0001, which will give the same network as a correlation threshold of 0.05. The threshold can be adjusted depending on the required level of complexity of the inferenced GRN. Figure 3 and Figure 4 show sub-graphs extracted from GLDS-7 root growth spaceflight and ground gene expression data, respectively, with a correlation threshold of 0.05. This threshold selects 11.3% of genes from the GLDS-7 dataset and 28.7% genes from the GLDS-120 dataset. These are weighted and directed sub-graphs extracted from the GLDS-7 spaceflight and ground GRNs, respectively. The weights are the correlation values (positive and negative) between the hub gene and the target gene, shown in Figure 3 and Figure 4. Negative interactions are important because they show an activation–inhibition two-way expression pattern. While positive correlations indicate shifting, scaling, or geometric pattern in gene expression data, negative correlations indicate opposite and complementary expression patterns, and self-negative regulation of transcription factors (TFs). Hence, we have extracted both maximal positive and negative correlations for constructing the GRNs. These gene interactions have not been validated by follow up experiments; however, they have been constructed from gene expression data obtained originally from experiments conducted in the ISS. 

**Figure 5:**
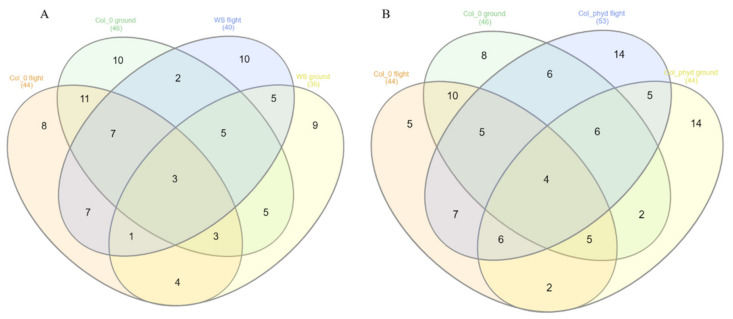
Venn diagrams showing the number of shared hub genes from the gene regulatory networks for *Arabidopsis thaliana*. (**A**) Between the WS and Col-0 ecotypes in spaceflight and ground control for both total light and dark environments. (**B**) Between Col-0 ecotype and Col-0 mutant *phyD* in spaceflight and ground control for both total light and dark environments.

Figure 5A shows the number of shared genes for Arabidopsis WS and Col-0 ecotypes between GRNs for spaceflight and ground data for both light and dark environments, respectively. Figure 5B shows the number of shared genes for the Arabidopsis Col-0 ecotype and Col-0 mutant *phyD* between GRNs for spaceflight and ground data for both light and dark environments, respectively. This helps us to make a secondary comparison between Col-0 ecotype with its mutant *phyD* and shows which hub genes are significantly represented in either case. Among the Arabidopsis ecotypes grown in spaceflight light environments, the GRN of Col-0 mutant *phyD* has 20 hub genes that are not shared with GRN of Col-0 ecotype grown in the same environment. The hub genes of Col-0 mutant *phyD* GRN overlap lesser with those of Col-0 than the overlap of the hub genes between WS and Col-0 GRNs in the light environment. The GRN of Col-0 mutant *phyD* in spaceflight light environment has the lowest number of shared hub genes. The *phyD* gene has a role in Red/Far-Red Light Sensing [48]. From our network analysis, we see that manipulation of a gene in an Arabidopsis ecotype can result in significantly different transcriptomic response that makes the biological organism to better adapt to the spaceflight environment [20].

**Figure 6:**
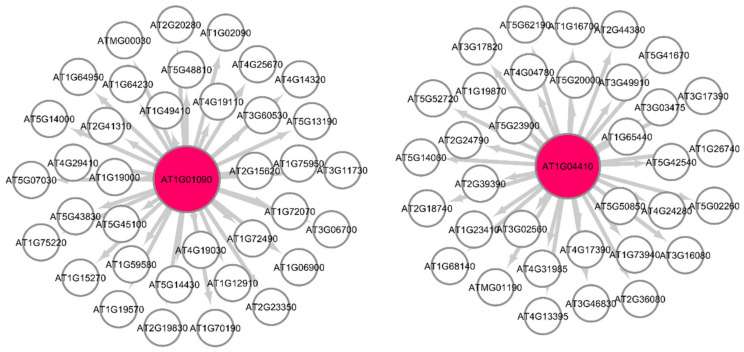
Gene regulatory sub-graph for the catabolic process in *Arabidopsis thaliana* Col-0 mutant *phyD* in spaceflight total light environment. The hub genes are the red circles representing the strong interactions with differentially expressed genes. The target (authority) genes are white circles.

Figure 6 shows the Pearson correlation gene regulatory sub-graphs extracted from GRNs of the GLDS-120 dataset for the catabolic process for the Arabidopsis WS ecotype root growth in spaceflight light environment. The number of hub genes in the GRN representation can be controlled by suitably adjusting the threshold value. The lower the threshold, larger number of hub genes can be detected and vice versa. In all our networks, we have used a nominal correlation threshold value of 0.05. The original networks have more than 600 genes involved in regulatory pathways. The GSEA of these hub genes present in spaceflight microgravity and ground control GRNs for WS, Col-0, and Col mutant *phyD* ecotypes grown in different light environments are presented below. 

### 3.1. Gene Set Enrichment Analysis (GSEA)

The hub genes, shared genes for spaceflight and ground environments, and their gene ontology from the GeneLab GLDS-7 root growth datasets are given in Table 2. The ShinyGO tool (http://bioinformatics.sdstate.edu/go/, accessed on 5 February 2021) is used to perform GSEA of the hub genes for the GeneLab GLDS-7 root growth GRN. The hub genes for spaceflight microgravity and ground overlap considerably and are involved in molecular functions, biological processes, and oxidation-reduction process. They enable NADH dehydrogenase activity, and are involved in NAD and quinone binding. Contrary to GLDS-7 GRN functions, the GRNs constructed for the *Arabidopsis* WS, Col-0, and Col mutant *phyD* show significantly different regulations in spaceflight in total light. These GRNs do share genes with ground control GRNs, but have other genes that show significant transcriptions in spaceflight microgravity, and these genes are not shared by the ground GRNs. Hence, these genes can be considered to be affected by spaceflight microgravity alone. These genes are related to biological processes such as DNA integration, the RNA-dependent DNA biosynthetic process, and the DNA metabolic process. We performed a GSEA analysis comparing hub genes for the WS wild ecotype and Col-0 ecotype. The hub genes for the WS ecotype are associated with biological processes such as the catabolic process, response to abiotic stimulus, localization, multicellular organismal process, developmental process, and anatomical structure development. The hub genes for the Col-0 ecotype perform similar functions to WS ecotype such as the catabolic process, regulation of metabolic process, positive regulation of biological process, cellular response to stimulus, signaling, multicellular organismal process, developmental process, and anatomical structure development. The shared hub genes between the Col-0 ecotype and Col-0 mutant *phyD* perform functions such as regulation of metabolic process, multicellular organismal process, developmental process, positive regulation of biological process, anatomical structure development, and catabolic process. The non-shared hub genes of *phyD* in spaceflight microgravity perform significant functions such as the cellular component organization of biogenesis, response to abiotic stress, and response to external stimulus.

#### 3.1.1. GSEA for Common Hub Genes

Eight common hub genes between the spaceflight and ground environments have been identified, as shown in Table 2. Using g:Profiler, we found that these genes, including the gene ATMG00600 (ORF 106C), are uncharacterized mitochondrial protein and their function is DNA integration. AT1G04080 is a shared gene between the Col-0 and WS ecotypes, which encodes a U1 small nuclear ribonucleoprotein (snRNP) factor involved in alternative splicing. AT1G02930 and AT1G04980 are the shared genes between the Col-0 and Col-0 mutant *phyD*, which are involved in the defense response to bacterium, glutathione metabolic process, response to cadmium ion, response to oxidative stress, water deprivation, heat, response to unfolded protein, and ubiquitin pathway. The Pearson and LASSO methods have led to the identification of signature sub-networks that show the interaction across several important metabolic and signaling processes, and Transcription Factors (TFs) for *Arabidopsis* growth in spaceflight microgravity. Both methods were able to identify the transcription factors AT1G06040, AT1G01720, and AT1G04850. AT1G06040 encodes salt tolerance protein and AT1G01720 is involved in the cellular response to hypoxia and has a DNA binding TF activity. The TF AT1G04850 is a ubiquitin-associated (UBA)/TS-N domain-containing protein, and its function is zinc ion binding. From the GRNs, we can identify genes with the maximum degree (hub genes) that regulate a larger number of authority (target) genes. 

#### 3.1.2. Gene Ontology of Transcription Factors (Genes) in Spaceflight

Thirty-eight hub genes with transcriptional abundance in spaceflight have been identified from the total light environment gene expression datasets of GLDS-120. Their ATG number, gene name, and gene ontology are presented in Supplementary Table S1. These are hub genes, as their transcriptional responses are abundant only in spaceflight microgravity network and are not found to be hub genes in the corresponding terrestrial GRNs. Hence, these genes are functioning only in spaceflight microgravity conditions. From their ontology, it is evident that they are responsible for important functions necessary for the plant’s growth in an altered environmental setup compared to the terrestrial environment. The plant can sense the changes in abiotic stressors such as gravity, oxygen, and radiation and can step up transcriptional regulation to adapt to the new environment successfully. The transcription functions of these genes are chiefly cell wall biosynthesis, protein transport, stress responses, catabolic processes, auxin, and protein regulation. It is also significant that these genes were identifiable only from the GLDS-120 dataset, which had a different lighting environment in spaceflight. Through novel graph theoretic methods applied to GRNs of *Arabidopsis*, we can explain phenomenological traits that have been observed in spaceflight vis-à-vis on the ground. 

### 3.2. Topological and Spectral Analyses of Gene Regulatory Networks

Topological and algebraic analyses of the GRNs are given in Table 3. The WS ecotype in total light spaceflight environment has the largest spectral gap, which indicates that this network has the highest performance with stable synchronization, rapid convergence, mixing, expansion, and diffusion [47]. Random walks propagate more easily in networks with a large spectral gap. The large spectral gap has also made several algorithms we use tractable for such massively large GRN and indicates that in technical terms, these GRNs are highly sparse. This corroborates with results from other studies that have shown that the WS genotype increases the transcriptional cost of spaceflight adaptation [20]. The diameter of this network is 6, indicating that it has longer gene signaling pathways, and the hub genes of this network regulate about 1000 target genes. The WS and Col-0 ecotypes in the ground light environment show topological similarities with the same shortest path length, and the two networks show regulation of about 45 target genes. 

We show, with our spectral analysis methods, through the spectral estimates on the Cheeger constant that our networks are sparse, that the HITS algorithm works much faster, and we have been able to identify hubs and authority nodes (genes) in the networks. Rather than just the degree distribution, hubs and authority nodes are computed iteratively with newer algorithms considering weights using its implementation in the software SAGEMATH. Improved algorithms exist, e.g., [35], and some of Kleinberg’s works.

### 3.3. Arabidopsis Root Growth and Cell Wall Biosynthesis in Spaceflight Microgravity

**Figure 7:**
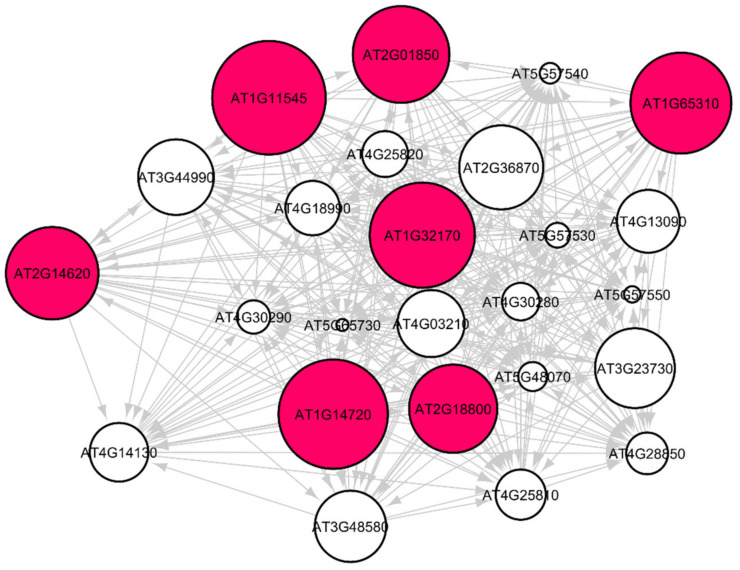
Gene regulatory sub-graph involving root growth genes in *Arabidopsis thaliana* Col-0 mutant *phyD* in spaceflight total light environment. The hub genes are the red circles representing the strong interactions with differentially expressed genes. The target (authority) genes are white circles. The hub genes that have a higher degree distribution are larger in size.

**Figure 8:**
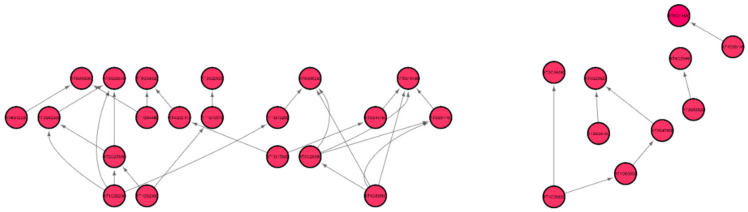
Gene regulatory sub-graph for the process of cell wall biosynthesis in *Arabidopsis thaliana* WS ecotype in spaceflight total light environment.

Out of the 33 root-growth related genes of *Arabidopsis*, we have identified five significant hub genes listed in Table 4 to have higher regulations in spaceflight. Figure 7 shows the subgraph that is extracted from the original Pearson correlation GRN by thresholding the genes with higher correlation values and involved in the cell wall biosynthesis metabolic process that relates the co-expression of these genes in spaceflight microgravity for the *Arabidopsis* Col mutant *phyD* ecotype. These five hub genes have higher regulations only in the spaceflight subgraph for the cell wall response process and not in the ground subgraph. These genes are over-represented in the spaceflight responsive transcriptome because they regulate plant growth in spaceflight microgravity and enable adaptation of the plant to the altered environment. Similarly, comparing the GRNs generated by the LASSO method for *Arabidopsis* WS ecotype in spaceflight and ground environments, the hub genes that are involved only in spaceflight for cell wall biosynthesis have been identified and listed in Supplementary Table S2. Figure 8 shows the subgraph for the cell wall biosynthesis process for *Arabidopsis* WS ecotype in spaceflight microgravity, which is also extracted similarly by thresholding the correlation values. These genes encode NSEs and have higher transcriptional regulation in spaceflight, as they were not found to be hubs in the GRNs for the terrestrial datasets. These enzymes catalyze a series of biochemical reactions and are involved in building cell wall polysaccharides. The origin and divergence of these enzymes is not known, and it is interesting to note that they are significantly transcribed indicative of higher sugar diversity in spaceflight microgravity. The higher regulations of these genes may be the reason for the phenotypic skewing or waving effect found in the roots, as well as other parts of the plant in spaceflight. The differential transcriptions of NSEs indicate that they regulate developmental, metabolic, and stress-related responses in the spaceflight microgravity environment.

## 4. Conclusions

Graph-based network analysis methods and results have been presented for transcriptomic regulatory GeneLab *Arabidopsis* datasets. Previous studies were at the transcriptomic level. In this article, we have built gene regulatory networks for different ecotypes (via Pearson Correlation and LASSO methods), carried out both topological and algebraic network studies (the novel HITS algorithm for weighted and directed graphs to identify hubs and authority nodes), computed the spectral gap for the whole GRN, leading us to potential conclusions about phenomena such as diffusion and expansion of the GRN, and studied global and local topological parameters, such as clustering, average path length, eccentricity, and degree distribution. Therefore, our research is more complementary to prior studies and is more rigorous. Our novel graph-theoretic approach isolated hub genes regulated significantly different in spaceflight microgravity from terrestrial gene expressions. The GRN for the WS ecotype in spaceflight has the highest spectral gap, indicating that it has the highest transcriptional cost in spaceflight. GRN analysis showed that there are 38 genes with higher transcriptions in spaceflight microgravity and are involved in vital molecular and biological functions for the survival and growth of the plant in spaceflight.

While previous research has identified altered spaceflight transcriptions for cell wall remodeling, we have identified hub genes with higher transcriptional activity and that are involved in the cell wall response process only in spaceflight GRN; these hub genes are essentially metabolic phenotypes. The network analysis has detected xyloglucan endo-trans glycosylase/hydrolases (*XTHs*) that are cell wall modifying enzymes mediating vital elongation growth of *Arabidopsis* in spaceflight. The *XTHs* act on the cell wall, making it more extensible, resulting in cellular expansion and the skewing phenomenon of the roots in spaceflight microgravity. 

Through advanced network analysis, we can shorten the gap between genotype to phenotype analysis by identifying primary metabolites or metabolic phenotypes involved in the growth of plants in spaceflight for bioregenerative life support. In the absence of mass spectroscopy, hyperspectral imaging methods in the ISS, and lack of plant physiological measurements, genome to phenome network analysis offers an alternative for identifying primary metabolic phenotypes. The spaceflight response of these metabolites can be further correlated with phenotypic data extracted from images and metabolomics datasets acquired from experiments conducted in the ISS or from similar experiments on the ground. Phenotypic data will enable novel trait correlations employing genome to phenome network analysis.

## Figures and Tables

**Table 1:** Hub genes and target authority genes for 13 hub genes with their correlation value, t-statistics, and *p*-value.

Interactor Gene A	Interactor Gene B	Correlation	t-Statistics	*p*-Value
AT1G03430	AT1G11185	−0.95787	−4.716511	0.04213
AT1G03430	AT1G12390	0.984801	8.018483	0.0152
AT1G03430	AT1G17170	−0.98068	−7.090435	0.01932
AT1G03430	AT1G17180	0.937057	3.795227	0.06294
AT1G03430	AT1G21140	0.454929	0.722456	0.54507
AT1G03430	AT1G24240	0.993113	11.98728	0.00689
AT1G03430	AT1G26470	0.98765	8.914699	0.01235
AT1G03430	AT1G27130 AT1G27140	0.998482	25.63509	0.00152
AT1G03430	AT1G27570	−0.95498	−4.552277	0.04502
AT1G03430	AT1G30360	0.9732	5.985038	0.0268
AT1G03430	AT1G32460	0.4588	0.730233	0.5412
AT1G03430	AT1G34180	−0.99271	−11.64964	0.00729
AT1G03430	AT1G34844	0.947816	4.204318	0.05218

**Table 2:** Hub genes from the Pearson Correlation GRN and HITS method and shared hub genes for GLDS 7 for *Arabidopsis thaliana* root growth in spaceflight and ground control with their gene ontology.

*Arabidopsis* Root	ATG Number	Enriched Sene Set (Biological Process)
Root growth spaceflight hub genes	ATMG00880 ATMG00840 ATMG00660	Part of mitochondrion; Enables molecular function; Involved in biological process
Root growth ground control hub genes	ATMG00720 ATMG01040 ATMG01020 ATMG00580	Part of mitochondrion Enables molecular function; Involved in biological process; ATP synthesis coupled electron transport; Involved in oxidation-reduction process
Shared hub genes for root growth in spaceflight and ground control	ATMG00860 ATMG00510 ATMG00750 ATMG00740 ATMG00720 ATMG00600 ATMG00680 ATMG00820	Part of mitochondrion Enables molecular function; Involved in biological process; Enables NADH dehydrogenase activity; Part of mitochondrial respiratory chain complex 1; Involved in oxidation-reduction process; Enables NAD binding; quinone binding

**Table 3:** Topological and spectral analysis for *Arabidopsis* (AT) spaceflight and ground gene regulatory networks.

Dataset	Spectral Gap	Average Clustering Coefficient	Average Shortest Path Length (Distance)	Diameter (Maximum Eccentricity)	Authority Genes (Outdegree)
AT root spaceflight	39.228	0.00897	1.452	3	95
AT root ground	59.96	0.00315	1.2017	3	166
AT WS spaceflight light	72.9	0.01272	1.89	6	1237.04
AT WS ground light	0.45	0.01788	1.11	2	45.63
AT WS spaceflight dark	3.99	0.01996	1.069	2	50.41
AT WS ground dark	2.32	0.02011	1.069	3	43.36
AT *phyD* spaceflight light	0.79	0.01000	1.036	2	35
AT *phyD* ground light	0.22	0.01996	1.069	2	50.41
AT *phyD* spaceflight dark	0.001	0.01219	1.146	2	38.24
AT *phyD* ground dark	3.15	0.0219	1.125	2	53.29
AT Col-0 spaceflight light	0.13	0.0212	1.107	2	40.26
AT Col-0 ground light	4.00	0.0146	1.113	2	44.28
AT Col-0 spaceflight dark	0.20	0.0224	1.066	1	29.09
AT Col-0 ground dark	2.67	0.0187	1.076	1	33.42

**Table 4:** Root growth hub genes with transcriptional abundance in spaceflight microgravity and their most prominent gene ontology.

ATG Number	Hub Gene Name/Description	Enriched Gene Set (Biological Process)
AT2G36870	*XTH32*	Involved in cellular glucan metabolic process
AT2G18800	*XTH21*	Involved in cell wall organization
AT3G44990	*XTH31*	Involved in glucanase activity
AT3G23730	*XTH16*	Involved in the metabolic process
AT2G14620	*XTH10*	Involved in cellular glucan metabolic process

## Data Availability

https://www.genelab.nasa.gov/.

## Supplementary Materials

The following are available online at https://www.mdpi.com/2073-4425/12/3/337/s1, The genelab datasets are available at http://www.genelab.nasa.gov/. Table S1: Hub genes differentially and significantly expressed only in spaceflight microgravity networks for each of the ecotypes, with their gene name and most prominent gene ontology; Table S2: Hub genes related to cell wall biosynthesis processes with transcriptional abundance in spaceflight microgravity, gene name and their most prominent gene ontology.
