# Supplementary material for: Network Analysis of Gene Transcriptions of Arabidopsis thaliana in Spaceflight Microgravity
Source: Genes (Basel). 2021 Feb 25;12(3):337. doi: 10.3390/genes12030337 (PMC7996555; doi:10.3390/genes12030337)
Supplement: Supplementary file 1 [file genes-12-00337-s001.pdf]

Supplementary Table S1. Hub genes differentially and significantly expressed only in spaceflight microgravity networks for each of the ecotypes, with their gene name and most prominent gene ontology.

Ecotype	ATG number	Gene name/Description	Enriched gene set (biological process)
Col_*phyD*	AT1G02140	Protein mago	Part of cytoplasm, enables protein binding, involved in RNA splicing, mRNA processing
	AT1G06400	Ras-related protein RABA1a	Involved in cell wall biogenesis, protein transport, response to auxin, enables GTP binding
	AT1G05620	Probable uridine nucleosidase 2	Enables purine nucleoside activity
	AT1G04250	Auxin responsive protein	Involved in auxin-activated signaling pathway
	AT1G05720	Sep15_SelM domain-containing protein	Enables oxidoreductase activity
	AT1G06040	Encodes salt tolerance protein (STO) which confers salt tolerance to yeast cells.	Enables protein, DNA, zinc ion binding, response to karrikin
	AT1G02130	This small GTP-binding protein is required in ER to Golgi transportation.	nuclear-transcribed mRNA catabolic process
	AT1G04690	Putative K+ channel	Involved in potassium ion transport
	AT1G06900	Nardilysin like	Enables metal ion binding
	AT1G05890	E3 ubiquitin-protein ligase AR15	Involved in protein ubiquitination
	AT1G01090	Pyruvate dehydrogenase E1 component subunit alpha-3, chloroplastic	Involved in acetyl-CoA biosynthetic process from pyruvate
	AT1G04040	At1g04040/F21M11_2	Involved in dephosphorylation
	AT1G06110	F-box protein SKIP 16	Enables protein binding
	AT1G02816	F22D16.19 protein	Enables molecular function

	AT1G06570	4-hydroxyphenylpyruvate dioxygenase	Involved in tyrosine catabolic process
	AT1G04850	UBA domain-containing protein	Part of extracellular region
	AT1G04750	Vesicle associated membrane protein	Enables protein binding
	AT1G02090	COP9 signalosome complex subunit 7	nuclear-transcribed mRNA catabolic process
	AT1G03900	Member of NAP protein family	nuclear-transcribed mRNA catabolic process
	AT1G06670	nuclear DEIH-box helicase (NIH) encoding a putative RNA and/or DNA helicase	GINS complex essential to the DNA replication process in the cells of eukaryotes; nuclear-transcribed mRNA catabolic process
	AT1G04820	Encodes an alpha tubulin isoform that is expressed in roots, leaves and flowers.	GINS complex; nuclear-transcribed mRNA catabolic process
Col_0	AT1G04750	vesicle-associated membrane protein 7B (At VAMP7B) mRNA,	Cellular component organization or biogenesis
	AT1G06570	4-hydroxyphenylpyruvate dioxygenase	Involved in tyrosine catabolic process
	AT1G04250	Auxin responsive protein	Involved in auxin-activated signaling pathway
	AT1G04850	UBA domain-containing protein	Part of extracellular region
	AT1G04750	Vesicle-associated membrane protein 721	Enables protein binding
	AT1G01300	Aspartyl protease protein family 2	Enables hydrolase, peptidase activity
	AT1G05890	Ubiquitin-protein ligase AR15	nuclear-transcribed mRNA catabolic process

	AT1G01160	Arabidopsis thaliana GRF1-interacting factor 2 (GIF2) mRNA	Enables protein binding
	AT1G06700	PTl1-like tyrosine-protein kinase 1	Enables kinase, transferase activity
WS	AT1G06550	Encodes salt tolerance protein (STO) which confers salt tolerance to yeast cells	Cellular component organization or biogenesis<br>Enables protein, DNA, zinc ion binding, response to karrikin
	AT1G04690	Putative K+ channel	Involved in potassium ion transport
	AT1G01090	Pyruvate dehydrogenase	Involved in the oxidation-reduction process
	AT1G05260	Encodes a cold-inducible cationic peroxidase that is involved in the stress response	Involved in cellular oxidant detoxification, desiccation and cold
	AT1G03870	Enables protein binding Fasciclin-like arabinogalactan protein 9	Involved in plant-type secondary cell wall biogenesis
	AT1G03900	Uncharacterized protein	Involved in vesicle-mediated transport
	AT1G02335	Germin-like protein	Involved in the regulation of root development

Supplementary Table S2. Hub genes related to cell wall biosynthesis processes with transcriptional abundance in spaceflight microgravity, gene name and their most prominent gene ontology.

Ecotype	ATG Number	Gene name/Description	Enriched gene set (biological process)
Col-0	AT2G27860	UDP-D-apiose/UDP-D-xylose synthase 1	Nucleotide-sugar biosynthetic process
	AT1G73250	GDP-L-fucose synthase 1	Oxidoreductase activity
	AT1G08200	UDP-D-apiose/UDP-D-xylose synthase 2	Involved in cell wall organization
	AT2G47650	UDP-glucuronic acid decarboxylase 4	Involved in D-xylose metabolic process

	AT2G28760	UDP-XYL synthase 6	Enables UDP-glucuronate decarboxylase activity
	AT3G14790	dTDP-glucose 4,6-dehydratase	Involved in auxin efflux
	AT1G63000	Bifunctional dTDP-4-dehydrorhamnose 3,5-epimerase/dTDP-4-dehydrorhamnose reductase	Enables dTDP-4-dehydrorhamnose 3,5-epimerase activity
	AT2G34850	Putative UDP-arabinose 4-epimerase 2	Enables UDP-glucose 4-epimerase activity
	AT1G17890	Putative GDP-L-fucose synthase 2	Involved in 'de novo' GDP-L-fucose biosynthetic process
	AT1G02000	Putative nucleotide sugar epimerase	Enables catalytic activity
	AT1G63180	UDP-glucose 4-epimerase	Enables UDP-arabinose 4-epimerase activity
	AT1G30620	UDP-arabinose 4-epimerase 1	Enables UDP-glucose 4-epimerase activity
	AT4G20460	Probable UDP-arabinose 4-epimerase 3	Enables UDP-arabinose 4-epimerase activity
	AT4G23920	UDP-glucose 4-epimerase 2	Enables UDP-glucose 4-epimerase activity
Col_phyD	AT1G12780	UDP-glucose 4-epimerase	Part of cytosol
	AT1G08200	UDP-D-apiose/UDP-D-xylose synthase 2	Involved in cell wall organization
	AT1G63000	Bifunctional dTDP-4-dehydrorhamnose 3,5-epimerase/dTDP-4-dehydrorhamnose reductase	Enables dTDP-4-dehydrorhamnose 3,5-epimerase activity

	AT3G53520	UDP-glucuronic acid decarboxylase 1	Enables UDP-glucuronate decarboxylase activity
	AT2G47650	UDP-glucuronic acid decarboxylase 4	Involved in D-xylose metabolic process
	AT4G12250	UDP-glucuronate 4-epimerase 5	Part of Golgi cisterna membrane
	AT5G39320	UDP-glucose 6-dehydrogenase 4	Part of cytosol
	AT1G26570	UDP-glucose 6-dehydrogenase 1	Involved in UDP-glucuronate biosynthetic process
	AT1G63180	UDP-glucose 4-epimerase	Enables UDP-arabinose 4-epimerase activity
	AT1G73250	GDP-L-fucose synthase 1	Oxidoreductase activity
	AT2G27860	UDP-D-apiose/UDP-D-xylose synthase 1	Nucleotide-sugar biosynthetic process
	AT1G02000	Putative nucleotide sugar epimerase	Enables catalytic activity
	AT1G64440	UDP-glucose 4-epimerase 4	Involved in carbohydrate metabolic process
WS	AT1G08200	UDP-D-apiose/UDP-D-xylose synthase 2	Involved in cell wall organization
	AT3G29360	UDP-glucose 6-dehydrogenase 2	Enables UDP-glucose 6-dehydrogenase activity
	AT1G64440	UDP-glucose 4-epimerase 4	Involved in carbohydrate metabolic process
	AT1G30620	UDP-arabinose 4-epimerase 1	Enables UDP-glucose 4-epimerase activity
	AT4G12250	UDP-glucuronate 4-epimerase 5	Part of Golgi cisterna membrane
	AT4G30440	UDP-glucuronate 4-epimerase 1	Involved in carbohydrate metabolic process
	AT1G17890	Putative GDP-L-fucose synthase 2	Involved in 'de novo' GDP-L-fucose biosynthetic process

	AT3G51160	GDP-mannose 4,6 dehydratase 2	Enables GDP-mannose 4,6-dehydratase activity
	AT1G02000	UDP-D-apiose/UDP-D-xylose synthase 2	Enables catalytic activity
	AT1G78570	UDP-4-keto-L-rhamnose-reductase RHM1	Involved in UDP-rhamnose biosynthetic process
